# Expanding the phenotypic variability of *MORC2* gene mutations: From Charcot‐Marie‐Tooth disease to late‐onset pure motor neuropathy

**DOI:** 10.1002/humu.24445

**Published:** 2022-08-18

**Authors:** Arnaud Jacquier, Shams Ribault, Michel Mendes, Nicolas Lacoste, Valérie Risson, Julien Carras, Philippe Latour, Aleksandra Nadaj‐Pakleza, Tanya Stojkovic, Laurent Schaeffer

**Affiliations:** ^1^ PGNM, Institut NeuroMyoGène Université Lyon1—CNRS UMR5261—INSERM U1315 Lyon France; ^2^ Centre de Biotechnologie Cellulaire, CBC Biotec CHU de Lyon—HCL groupement Est Bron France; ^3^ Service de Médecine Physique et de Réadaptation Hôpital Henry Gabrielle, Hospices Civils de Lyon Saint‐Genis‐Laval France; ^4^ Service de Neurologie Centro Hospitalar Trás‐os‐Montes e Alto Douro Vila Real Portugal; ^5^ Unité fonctionnelle de neurogénétique moléculaire CHU de Lyon—HCL groupement Est Bron France; ^6^ Centre de Référence des maladies Neuromusculaires Nord/Est/Ile‐de‐France, Service de Neurologie Hôpitaux Universitaires de Strasbourg Strasbourg France; ^7^ Institut de Myologie Hôpital Pitié‐Salpêtrière Paris France

**Keywords:** Charcot‐Marie‐Tooth, CMT2z, DIGFAN syndrome, MORC2, motoneuron, SMA‐like, spinal muscular atrophy

## Abstract

*MORC2* gene encodes a ubiquitously expressed nuclear protein involved in chromatin remodeling, DNA repair, and transcriptional regulation. Heterozygous mutations in *MORC2* gene have been associated with a spectrum of disorders affecting the peripheral nervous system such as Charcot‐Marie‐Tooth (CMT2Z), spinal muscular atrophy‐like with or without cerebellar involvement, and a developmental syndrome associated with impaired growth, craniofacial dysmorphism and axonal neuropathy (DIGFAN syndrome). Such variability in clinical manifestations associated with the increasing number of variants of unknown significance detected by next‐generation sequencing constitutes a serious diagnostic challenge. Here we report the characterization of an in vitro model to evaluate the pathogenicity of variants of unknown significance based on *MORC2* overexpression in a neuroblastoma cell line SH‐EP or cortical neurons. Likewise, we show that *MORC2* mutants affect survival and trigger apoptosis over time in SH‐EP cell line. Furthermore, overexpression in primary cortical neurons increases apoptotic cell death and decreases neurite outgrowth. Altogether, these approaches establish the pathogenicity of two new variants p.Gly444Arg and p.His446Gln in three patients from two families. These new mutations in *MORC2* gene are associated with autosomal dominant CMT and with adult late onset proximal motor neuropathy, further increasing the spectrum of clinical manifestations associated with *MORC2* mutations.

## Graphical abstract

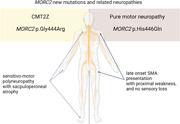

## 1 INTRODUCTION

*MORC2* gene encodes a member of the Microrchidia (MORC) protein superfamily well conserved among higher Eukaryotes (Inoue, 1999). *MORC2* gene is ubiquitously expressed, with the highest expression in testis, ovary, and brain. RT‐PCR enzyme‐linked immunoassay shows moderate expression in lungs, kidneys, liver, and heart, and an even lower expression in skeletal muscle, pancreas, and spleen (Nagase et al., 1998). Transcriptomic analysis in mice indicates that *Morc2* expression undergoes spatiotemporal regulation in the brain with a peak expression at the earlier developmental stages followed by a progressive decrease during aging suggesting an important role in the development of the nervous systems (Sancho et al., 2019). MORC2 protein was initially shown to regulate transcriptional repression, invasiveness, and lipogenesis in cancer cells (Liao et al., 2017; Sánchez‐Solana et al., 2014; Shao et al., 2010). MORC2 was then shown to relax chromatin and facilitate DNA double‐strand break repair via a DNA‐dependent ATPase activity (Li et al., 2012). More recently, *MORC2* gene was shown to be involved in epigenetic silencing by the human silencing hub (HUSH) complex (Douse et al., 2018; Tchasovnikarova et al., 2017).

Heterozygous mutations in *MORC2* gene are associated with a spectrum of disorders affecting the peripheral nervous system. Most clinical manifestations of *MORC2* mutations are associated with an axonal form of Charcot‐Marie‐Tooth disease (CMT2Z; MIM# 616688) (Albulym et al., 2016; Laššuthová et al., 2016; Semplicini et al., 2017; Sevilla et al., 2016; Sivera et al., 2021; Zhao et al., 2016). The main clinical features of the disease encompass slowly progressive distal weakness, muscle atrophy associated with sensory impairment, typically occurring during childhood or adolescence. However, some phenotypic variability exists among *MORC2* mutated CMT patients, some of them exhibiting hearing loss (Albulym et al., 2016; Sevilla et al., 2016), pyramidal signs and seizures (Albulym et al., 2016). Occasionally, *MORC2* mutations produce early‐onset spinal muscular atrophy‐like (SMA‐like) phenotypes characterized by proximal muscle and atrophy without sensory loss with or without diaphragmatic palsy (Schottmann et al., 2016; Zanni et al., 2017), microcephaly (Sevilla et al., 2016; Zanni et al., 2017), or cerebellar atrophy (Schottmann et al., 2016). Recently, 20 individuals with heterozygous *MORC2* mutations were shown to develop a neurodevelopmental syndrome associated with intellectual disability, growth retardation, facial dysmorphism, and axonal neuropathy (DIGFAN; MIM# 619090) (Guillen Sacoto et al., 2020). The pathophysiological mechanisms underlying such phenotypic variability cannot be correlated to specific mutated amino acids and remain unclear. Furthermore, with the increasing number of variants of unknown significance identified by NGS, clinicians need to develop a functional assay to decipher between pathological variants and polymorphism.

Here, to evaluate the pathogenicity of *MORC2* variants of unknown significance, we have developed an in vitro system based on the overexpression of wild‐type (WT) or mutant MORC2 proteins in a neuroblastoma cell line SH‐EP and in primary cortical neurons. By quantifying survival and apoptosis over time, we show significant differences between WT and well‐characterized MORC2 mutants which allow to predict the pathogenicity of two new variants c.1330G>A (p.Gly444Arg) and c.1338C>A (p.His446Gln) of the *MORC2* gene respectively associated with an autosomal dominant form of CMT and with adult late onset proximal motor neuropathy.

## 2 MATERIALS AND METHODS

### 2.1 Patients and mutation analysis

Clinical evaluation and biological samples were obtained after informed written consent and were secured in accordance with the protocol approved by national ethic committees. Routine biological analyses were performed on the three patients, as well as nerve conduction studies (NCS) and electromyogram (EMG) studies.

Patient from family 2 performed whole‐body muscle magnetic resonance imaging (MRI) and was screened for mutations in the *SMN1, HSPB1, HSPB3*, and *HSPB8* genes. Additionally, whole‐exome sequencing was performed. Exome enrichment was done with the SeqCap EZ MedExome Target Enrichment Kit, and sequencing was done using the NextSeq. 500 sequencing system following the manufacturers' instructions.

Patient II4 from family 2 performed a next‐generation sequencing‐based gene panel that includes 76 genes. A search for mutations using Sanger sequencing of the codified regions of the *MORC2* gene was performed in patient III5.

### 2.2 Plasmids

MORC2‐flag fusion protein was realized by Genescript by cloning *MORC2* ORF complementary DNA (NM_001303256.2) in pcDNA3.1‐C‐DYK plasmid. Mutagenesis was done by site‐directed mutagenesis following the manufacturer recommendation (QuikChange II Site‐Directed Mutagenesis Kit—Agilent) and clones were validated by sequencing. *MORC2*‐flag variants were subcloned in pCAGIG vector (addgene #plasmid 11159; Matsuda & Cepko, 2004). *MORC2*‐flag (3kb) variants were amplified by high fidelity PCR (Q5, New England BioLabs) and cloned using XhoI/NotI. Clones were validated by sequencing the full coding sequence.

### 2.3 Cell culture

Human neuroblastoma cell line SH‐EP (RRID:CVCL_0524; an epithelioid subclone of the human NB line SK‐N‐SH) was used in this study. Cells were defrosted and cultured in a modified Dulbecco's modified Eagle medium (DMEM) culture medium (DMEM medium with 10% fetal bovine serum and 1% penicillin/streptomycin) and were transfected using JetPRIME following manufacturer recommendations (Polyplus transfection). Cortical neuron culture was done as described previously in Jacquier et al. (2009). Briefly, embryonic mice at E15.5 days of development were killed. The motor cortex was dissected and trypsinized. Cortical neurons were then transfected by electroporation using a cuvette electroporator (BTX ECM830) with the following parameters 220 V, 5 ms pulse length, 3 times at 1‐s interval. Electroporated neurons were plated in standard conditions (neurobasal plus medium + B27 plus + 2% horse serum + 1% penicillin/streptomycin).

### 2.4 Western blot

SH‐EP were plated in six well plate and transfected with the different plasmids using the JetPrime reagent (Polyplus transfection). Twenty‐four hours later, cells were washed in phosphate‐buffered saline (PBS) and extracted directly in 200 µl 1X Laemmli buffer (50 mM Tris HCl pH 6.8, 10% glycerol, 100 mM DTT, 2% SDS, bromophenol blue) supplemented with 20U of Benzonase (Merck Millipore) per wells to digest the DNA. After 15‐min incubation at room temperature (RT), totals extracts are boiled and loaded in an 8% sodium dodecyl sulfate–polyacrylamide gel electrophoresis, transferred on nitrocellulose membrane, blotted with the indicated antibodies, and revealed with the ECL clarity (BioRad).

### 2.5 Immuno fluorescent staining and quantification

Cells line or cortical neuron culture were fixed using 4% formaldehyde for 10 min at RT. Then, cells were washed in PBS and put 1 h in blocking buffer (PBS with 4% bovine serum albumin, 0.3% Triton X‐100, 0.1 M glycine) before primary antibody incubation overnight at 4°C. After three PBS washes, secondary antibodies were incubated in a blocking buffer for 1 h at RT, washed and mounted. The following antibodies were used. Mouse anti GFP at 1/1000 (sc‐9996; Santa Cruz); Rabbit anti‐Cleaved caspase‐3 Asp175 at 1/400 (#9661; Cell Signaling Technology); Rabbit anti FLAG M2 at 1/1000 (#14793; Cell Signaling Technology).

Images acquisitions were done using EVOS M5000 microscope (ThermoFisher Scientific) or LSM800 confocal microscope (Zeiss). Images were analyzed using ImageJ software or Metamorph software. In SH‐EP experiments, quantification of GFP positive cells were done on at least 80 images from at least 4 independent experiments. To overcome difference in transfection efficiency between conditions, survival was normalized by Day 1. To pool the data between independent experiments, survival was expressed in percentage of the control. Caspase 3 activation quantification was established by the percentage of caspase 3 activated positive cell in GFP positive cells expressing MORC2 and normalized at 100% for the control. In cortical neuron experiments, quantification of the soma number was done on confocal images using Metamorph software on at least 120 fields per condition from 4 independent experiments. The mean number of cortical neurons per field was normalized to the value at Day 1 and compared to the WT condition. The percentage of activated‐caspase 3 positive neurons in electroporated neurons (i.e., GFP positive) was quantified on 80 fields per condition from 4 independent experiments and normalized to the value obtained with WT MORC2. Neurite length for each neuron was automatically measured in confocal images using the specific plugin “neurite outgrowth” of the Metamorph software. Quantification was performed on 30 fields per experiment in 4 independent experiments at Day 5.

### 2.6 Statistical analysis

Each experiment was independently repeated at least three times. Data were analyzed with Excel (Microsoft) or Prism 5 (GraphPad Software Inc). Data from several groups showing normality and equal variance were analyzed with one‐way analysis of variance followed by Dunnett's multiple comparison test. Otherwise, data that do not show a Gaussian distribution were analyzed with Kruskal–Wallis nonparametric test followed by Dunns post hoc test. Asterisks describes values levels of statistical significance as following: Non significant ns: *p* < 0.05; significant *p < 0.01; very significant **p< 0.0001; extremely significant ***p < 0.0001.

### 2.7 Ethical approval

All procedures performed in studies involving human participants were in accordance with the ethical standards of the institutional and/or national research committee and with the 1964 Helsinki declaration and its later amendments or comparable ethical standards. Informed consent was obtained from all individual participants included in the study. All procedures performed in studies involving animals were in accordance with the ethical standards of the institution or practice at which the studies were conducted.

## 3 RESULTS

### 3.1 The clinical features and ancillary exams

#### 3.1.1 Family 1

**Figure 1:**
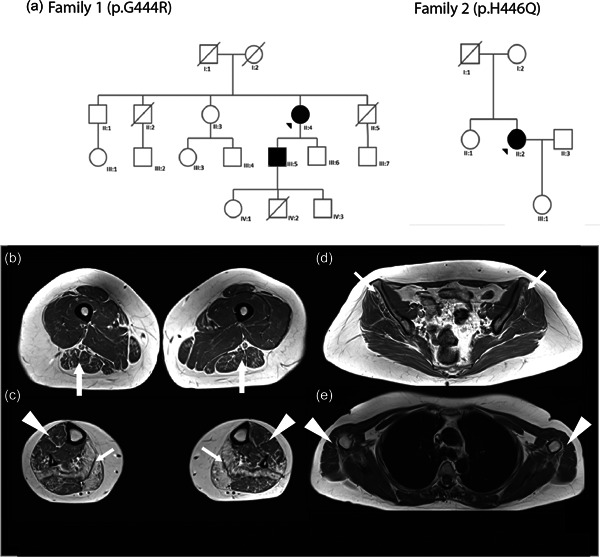
Pedigree of the two families. (a) Pedigree of families 1 and 2. Black arrows represent proband. (b–e). Axial T1‐weighted lower limbs muscle MRI scans from Patient F2: II2. Fatty infiltration was graded using Mercuri score (Mercuri et al., 2002) graded from 0 (*normal appearance*) to 4 (*total fatty replacement of the muscle*). (b) Legs: Atrophy and fat replacement (Mercuri Grade 4) prominently in solei and gastrocnemii medialis (arrows). Mild fatty infiltration in the tibialis anterior and peroneus lateralis (arrowheads), and gastrocnemius lateralis (Grade 2). (c) Thigh: Asymmetrical atrophy of the thigh more pronounced on the right side. Mild fatty infiltration observed in biceps femoris (arrows), semitendinosus, semimembranosus, and sartorius muscles. (Grade 2). (d) Pelvis girdle: Moderate the fatty degeneration of the gluteus minimus (arrows) and gluteus medius (Grades 2 and 1, respectively, for the right and left side). (e) Scapular girdle: Note the mild fatty infiltration of the deltoids (arrowheads). MRI, magnetic resonance imaging

Subjects II4 and III5 are the only affected members of a French nonconsanguineous family (Figure 1). Subject II4 reported an intentional tremor during adolescence and presented frequent falls and hand motor weakness from the age of 30 years. Over decades, the weakness has spread in the pelvic and scapular girdle, resulting in disability and wheelchair requirement by the age of 65 years. On examination, she presented severe muscular weakness in the distal lower limbs (1/5 on Medical Research Council [MRC] Scale for muscle strength) and distal upper limbs (2/5 on MRC scale). Proximal weakness was also noted, mostly in the deltoids, biceps brachialis, and psoas iliacus (2/5 on MRC scale), but also in the quadriceps and hamstring muscles (4/5 and 3/5 on MRC scale, respectively). Diffuse wasting and generalized areflexia were present. Sensory complaints were minor and characterized by a diminished vibratory sense in all four limbs. NCS showed severe motor and sensory axonal neuropathy (Table 1). The Creatine Kinase (CK) level was in the normal range (198 UI/L; normal > <200 UI/L). Her son (III5) developed an intentional tremor, hand motor weakness at the age of 20 years, and had walking difficulties and distal leg weakness from the age of 23 years. Neurological examination at 46 years showed distal muscular atrophy and severe distal weakness in lower limbs. Tibialis anterior and foot extensors were rated 1/5 whereas triceps was rated 2/5 on MRC scale). Mild proximal weakness in upper limbs was also noted (deltoids and biceps brachialis rated 4/5 on MRC scale) and the intrinsic hand muscles were weaker and rated 2/5 on MRC scale. Tendon reflexes were absent. No sensory impairment was noticed. NCS and EMG showed motor and sensory axonal neuropathy (Table 1).

**Table 1:** Summarized clinical and ancillary tests findings

	Family 1		Family 2
	II.4	III.5	II.2
Gender	Female	Male	Female
Age at first symptoms (years)	30	20	Early adulthood
Age at last examination (years)	66	46	46
Distal weakness^a^			
UL	++	++	+
LL	+++	+++	−
Distal atrophy^b^			
UL	++	++	−
LL	++	++	−
Proximal weakness^a^			
UL	++	+	−
LL	++	−	+
Foot deformity	−	−	Pes cavus
Motor performance at last visit	Wheelchair	Ankle‐foot orthoses. Independent ambulation but limited gait perimeter	Independent ambulation but limited gait perimeter
Distal sensory loss^c^	+	−	−
Spine deformity	+	−	−
Deep tendon reflex^d^ (UL/LL)	−/−	−/−	+/− (ankle)
Motor NCS^e^ (MNCV/CMAP)			
Median nerve	31.1/0.44	52.8/9.6	60/7.02
Ulnar nerve	41.1/1.22	48/14.6	67/7.60
Common peroneal nerve	26.7/0.38	38.5/0.5	53/8.14
Tibial nerve	28.2/0.8	37/5.5	NA/12
Sensory NCS^f^ (SNCV/SNAP)			
Median nerve (Transcarpal)	NA	51.3/0.69	55/124
Ulnar nerve (5th finger)	NR	NA	55/21.2
Radial nerve	NR	48.8/3.4	NA
Superficial peroneal nerve	NA	NA	37/26.4
Sural nerve	NR	NA	NA

Abbreviations: LL, lower limbs; NA, no data available; NCS, nerve conduction studies; UL, upper limbs.

^a^ (−) no weakness, (+) 4/5 on Medical Research Council (MRC) scale, (++) <4/5 on MRC scale, (+++) complete paralysis.

^b^ (−) no atrophy, (+) mild atrophy, (++) moderate or severe atrophy.

^c^ (−) normal, (+) mild, (++) profound.

^d^ (+) normal, (+/−) decreased, (−) areflexia.

^e^ Motor nerve conduction studies (NCS). MNCV: motor nerve conduction velocity (m/s). CMAP: compound motor action potentials (mV); Muscles recorded: median: abductor pollicis brevis/ulnar: adductor digiti minimi/superficial peroneal: extensor digitorum brevis/tibial: tibialis posterior.

^f^ Sensory nerve conduction velocity (NCS). Sensory conduction velocities (m/s). SNAP: sensory nerve action potential (µV). NA = not available; NR = not recordable.

#### 3.1.2 Family 2

Subject II2 is the only known affected member of a Portuguese nonconsanguineous family (Figure 1) although her father also experienced cramps but there was no familial history otherwise and no consanguinity. She had no history of physical development delay. She reported cramps in the lower limbs and a tremor of the upper extremities in early adulthood. At 30 years, she displayed muscular weakness with walking impairment and difficulties climbing stairs. The disease slowly progressed, and at age 44, her clinical examination revealed distal weakness in the upper limbs (wrist extensor and interosseous scored as 4/5 on MRC scale) and a proximal motor deficit in the lower limbs (quadriceps, psoas, and hamstring muscles as 4/5 on MRC scale), with *pes cavus*. Jerk reflexes were all present except at the ankle. No pyramidal signs or cranial nerve impairments were observed. Electroneuromyography showed neurogenic features, in the forearms, and both proximal and distal in the lower limbs, with normal motor and sensory conduction (Table 1). CK level was mildly elevated: 258 UI/L (Normal <200 UI/L). A whole‐body muscle MRI was performed at 46 years of age, showing a severe, bilateral, and symmetric fatty substitution more apparent in gastrocnemius medialis and soleus muscles. In the thighs and pelvis, there was a mild fatty replacement of posterior thigh musculature and glutei (Figure 1b). In the upper limbs, there was also a mild wasting of biceps brachialis, and deltoids.

### 3.2 Molecular findings and bioinformatics analysis

Whole‐exome sequencing was performed for both families. The two affected individuals of the first family bear the same heterozygous mutation c.1330G>A (p.Gly444Arg) in the *MORC2* gene (NM_001303256.2; MIN# 616661). Patient II2 from family 2 bears the heterozygous mutation c.1338C>A (p.His446Gln) in the *MORC2* gene. Sequencing of the *MORC2* gene in her mother and sister's DNA (both asymptomatic) revealed no mutation. Her father was deceased hence no DNA was available.

**Figure 2:**
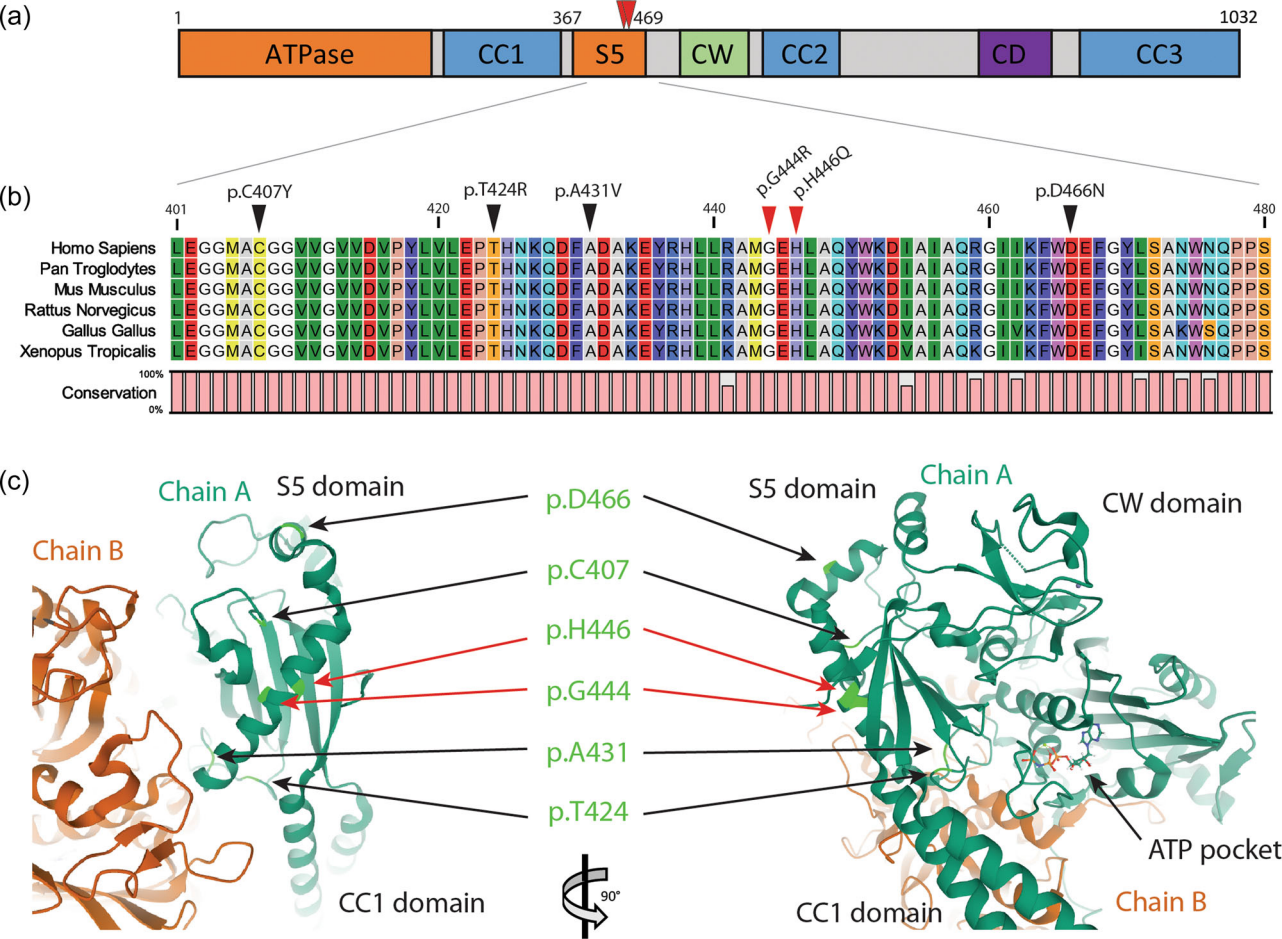
Bioinformatic studies of MORC2 mutations. (a) MORC2 protein (NP_001290185) predicted domains. An N‐terminal GHJL‐type ATPase domain together with a ribosomal protein S5 (RPS5; MIM# 603630) form the functional ATPase catalytic site. CC: coiled‐coil regions required for protein dimerization. CW: a zinc finger‐type CW domain allowing interaction with DNA. CD: a chromo‐like domain recognizing methylated histone proteins. (b) Multiple alignments of MORC2 orthologous genes selected from 401 to 480 amino acids in MORC2 human protein. Black arrows indicate known mutation in the area. Red arrows indicate mutation positions in patient families 1 and 2 describes here. Color amino acids follow RasMol color scheme according to traditional amino acid properties. Outer parts of a protein that are polar are visible (bright) colors and nonpolar residues darker. Conservation level is indicated by a bar plot. (c). Three‐dimensional structure of homodimeric human MORC2 focused on S5 transactivator domain showing the localization of mutated residue (light green) on chain A (dark green). MORC, member of the Microrchidia

MORC2 protein (NP_001290185) contains several predicted domains (Figure 2a). Interestingly, all known mutations affect the ATPase domain or its transducer S5 domain (Douse et al., 2018). Both mutations described here affect conserved amino acids in the S5 domain at positions 444 and 446 (Figure 2b). Neighboring mutations in the S5 domain are p.C407Y (Ando et al., 2017), p.Thr424Arg (Schottmann et al., 2016; Zanni et al., 2017), p.A431V (Ando et al., 2017), p.D466N (Semplicini et al., 2017). Interestingly, mutation p.Gly444Arg has been reported by Albulym, et al (Albulym et al., 2016) as a likely pathogenic variant without familial or functional evidence.

Bioinformatic prediction by HumVAr (PolyPhen‐2) identified mutations p.Gly444Arg and p.His446Gln as probably pathogenic with a score of 0.945 and 0.878 on a 0–1 scale, with a sensitivity of 0.65 or 0.71 and a specificity of 0.91 or 0.89, respectively. Likewise, the mutation taster software found that both mutations were pathogenic and were neither found in gnomAD (The Genome Aggregation Database) nor 1000G. Interestingly, the crystal structure of MORC2 was solved (Douse et al., 2018) allowing to map the mutations (Figure 2c) and predicting their impact on the 3D structure of the protein. This allowed the HOPE software to predict that the mutations were structurally damaging. Indeed, the arginine mutant residue in position 444 is bigger and introduces a charge in a buried residue which probably perturbs protein folding. The glutamine mutant residue at position 446 breaks a hydrogen bond between Histidine 444 and Alanine 442 residue and, therefore, destabilizes the alpha helix.

### 3.3 Protein construct and subcellular localization

A flag tag (also called DYK) (Figure S1A) was fused to the C‐terminal extremity of *MORC2* and an internal ribosome entry site and eGFP were added downstream (pCAGIG plasmid, addgene #11159). To check the subcellular localization of the MORC2‐flag fusion protein, SH‐EP neuroblastoma cells were transfected (Reddy et al., 1991) and murine primary cortical neurons were electroporated. Two days after transfection, the cells were fixed and MORC2 was stained with the anti‐Flag antibody. Nuclei were counterstained with DAPI (Figure S1B). In both cell types, MORC2 was detected in the nucleus.

Site‐directed mutagenesis was done on pCAGIG *MORC2*‐Flag to reproduce the mutations identified in the patients. As positive pathogenic controls mutations were introduced at the following positions of the *MORC2* coding sequence (NM_001303256.2): c.754 C>T leading to MORC2 p.Arg252Trp related to CMT2 (Sevilla et al., 2016), c.1271 C>G leading to MORC2 p.Thr424Arg related to SMA‐like (Schottmann et al., 2016; Zanni et al., 2017). Similarly, both variants of unknown significance were generated by mutagenesis at positions c.1330G>A leading to MORC2 p.Gly444Arg and c.1338C>A leading to p.His446Gln. By convention, these variants will be designated in the figures by their position (i.e., p.Gly444Arg as p444). To evaluate the expression of the protein, the same amount of each plasmid was transfected in SH‐EP and total proteins were solubilized in Laemmli buffer 24 h later and analyzed by western Western blot (Figure S1B). Ponceau Red staining revealed the total protein loaded whereas anti‐MORC2 or anti‐Flag antibodies were used to detect MORC2. Altogether, WT and mutant MORC2 proteins were expressed at the same level. Furthermore, immunofluorescence and confocal microscopy detected no difference in the subcellular localization of WT or MORC2 mutants (data not shown).

### 3.4 MORC2 mutants alter SH‐EP survival and trigger apoptosis

**Figure 3:**
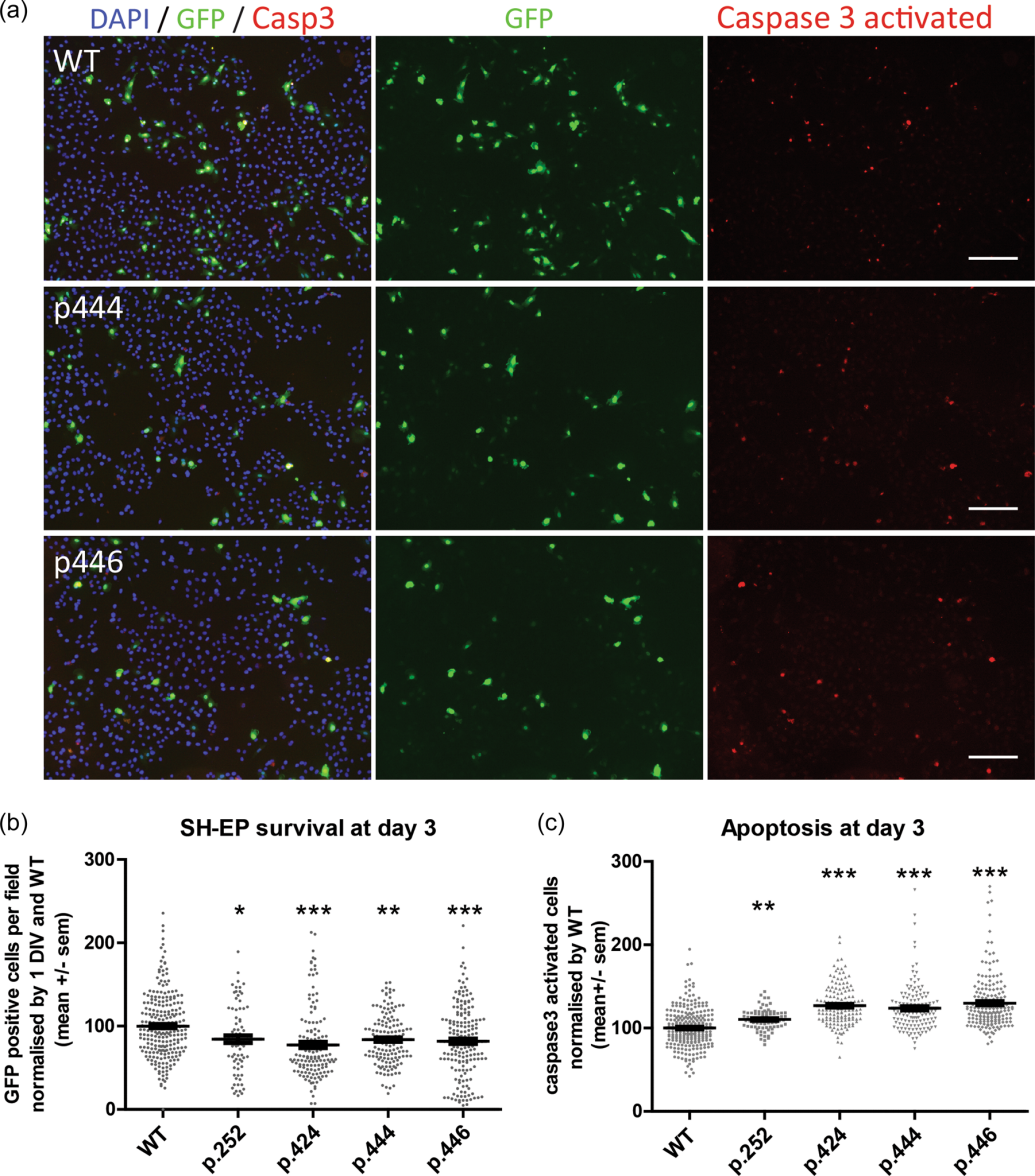
MORC2 mutants affect SH‐EP survival and apoptosis. (a) Microscope images of SH‐EP culture at 3 days in vitro after MORC2 transfection. Cell are counted stained for nuclei with DAPI (in blue). Apoptotic cells are stained with anti activated‐caspase 3 antibody (in red). Scale 100 µm. (b) SH‐EP survival quantification by counting eGFP positive cells in more than 75 fields from more than 3 independent experiments. Kruskal–Wallis test followed by Dunn's multiple comparison test. (c) Quantification of the percentage of activated‐caspase 3 positive cell at Day 3 and normalized by WT condition. Kruskal–Wallis test followed by Dunn's multiple comparison test. ns *p* < 0.05; *p < 0.01; **p < 0.001; ***p< 0.0001. p252 is p.Arg252Trp; p424 is p.Thr424Arg; p444 is p.Gly444Arg; p446 is p.His446Gln.

To determine the impact of *MORC2* variants on SH‐EP survival over time, transfected cells (GFP positive) were analyzed after 1, and 3 days in culture (Figure 3a). The cell number was attributed a value of 100% at Day 1. To evaluate survival, eGFP positive cells were counted at Day 3 and normalized to the number of cells at Day 1. At Day 3, all mutants caused a decrease in survival compared to control, including the variants p.Gly444Arg (84%) and p.His446Gln (82%) of unknown significance (Figure 3b).

Activation of the caspase 3 by proteolytic cleavage is a landmark of apoptosis‐mediated cell death. To evaluate if reduced survival induced by MORC2 mutants involved the induction of apoptosis, activated caspase 3 was detected by immunofluorescence at Day 3 of culture after transfection. Quantification of activated caspase 3 positive cells at Day 3 revealed a significant increase in all MORC2 mutants: p.Arg252Trp (110%), p.Thr424Arg (127%) or MORC2 p.Gly444Arg (124%), p.His446Gln (130%) compared to WT MORC2 (100%). Altogether, these results suggest that *MORC2* variants decrease cell survival through apoptosis.

### 3.5 Mutant MORC2 affect cortical neuron survival

**Figure 4:**
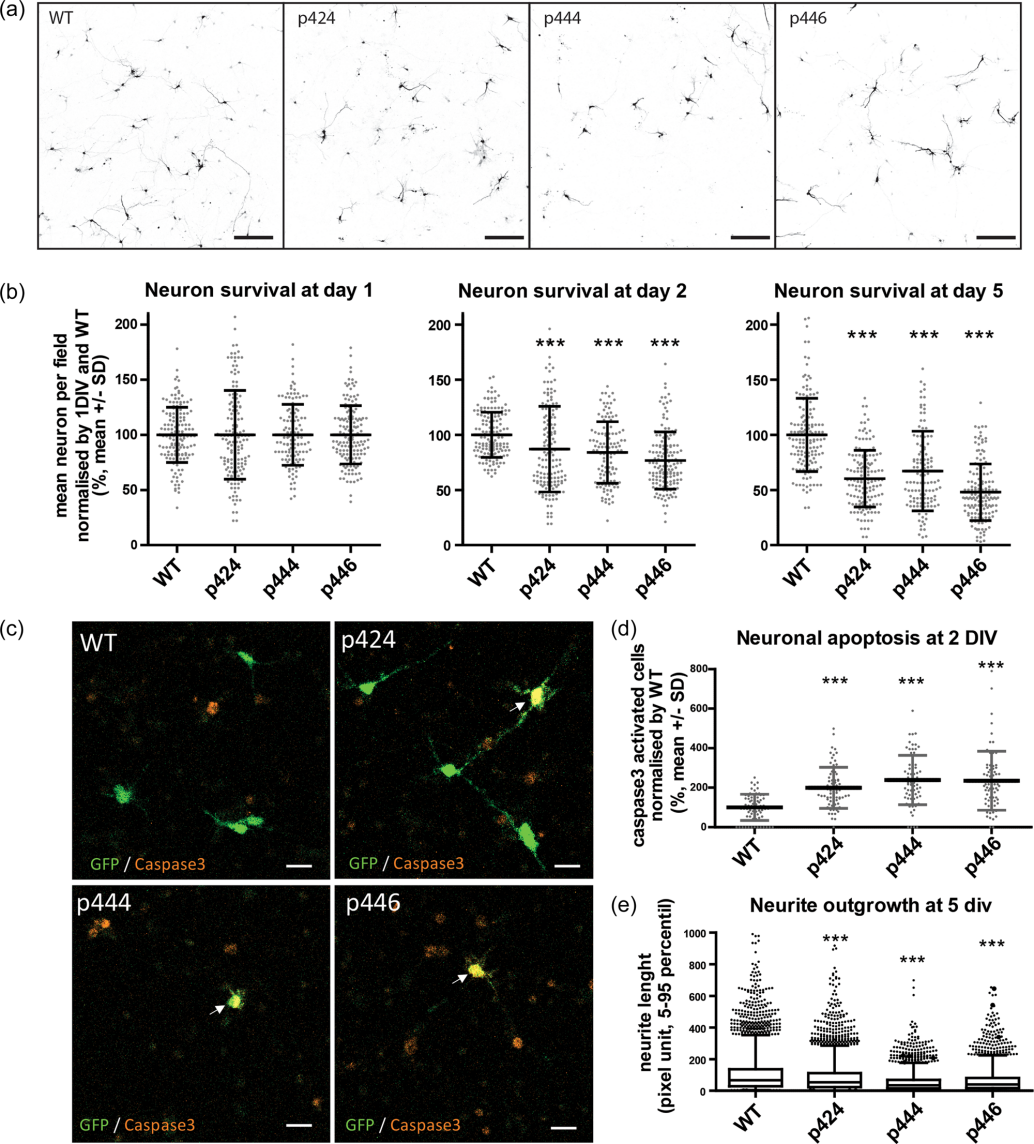
MORC2 mutants affect cortical neuron survival and neurite outgrowth. (a) Confocal images of eGFP cortical neuron at Day 5 revealing soma and neurites of neurons expressing MORC2 (scale bar: 200 µm). (b) Survival quantification of cortical neurons over time by counting eGFP soma in at least 120 fields per condition from more than four independent experiments. Kruskal–Wallis test followed by Dunn's multiple comparison test. ***p < 0.0001. (c) Confocal images of cortical neurons at Day 2 expressing MORC2 and eGFP counterstain for activated caspase 3 (in red). Scale 20 µm. (d) Quantification of the number of activated‐caspase 3 neurons per field at Day 2 normalized by WT condition (70 fields from 4 independent experiments). Kruskal–Wallis test followed by Dunn's multiple comparison test. ***p < 0.0001. (e). Box plot representation of the axon length distribution (at least 2500 neurons, from 120 fields, from 4 independent experiments) Kruskal–Wallis test followed by Dunn's multiple comparison test. p424 is p.Thr424Arg; p444 is p.Gly444Arg; p446 is p.His446Gln. MORC, member of the Microrchidia; WT, wild type

Since mutations in *MORC2* are associated with neurodegenerative disorders, we tested their effect in primary cells more relevant for to the pathophysiology of *MORC2*‐related disorders. Cortical neurons of the motor cortex, also called upper motor neurons, are easy to purify in large quantities and can be transfected by electroporation. To determine the impact of MORC2 mutants on the survival, cortical neurons were electroporated with WT, p.Thr424Arg, p.Gly444Arg or p.His446Gln MORC2 constructs immediately after dissociation and analyzed at Days 1, 2, and 5 (Figure 4a). Cortical neuron counting over time showed that a decrease in survival was already observed at Day 2 with the pathogenic mutation (p.Thr424Arg, 87%) and with the p.Gly444Arg (84%) and p.His446Gln (77%) variants compared to WT MORC2 (Figure 4b). Similar results were observed at Day 5 after electroporation with the pathogenic mutant p.Thr424Arg (60.34%) and the two variants of unknown significance p.Gly444Arg (67.19%) and p.His446Gln (47.97%) compared to the WT (100%), suggesting that these variants are pathogenic.

Since we observed increased apoptosis in SH‐EP cell expressing the MORC2 mutants, we investigated whether it was also the case in motor neurons. Activated‐caspase 3 staining was performed 2 days after electroporation (Figure 4c). The percentage of activated‐caspase 3 positive neurons in electroporated neurons (i.e., GFP positive) indicated that after 2 days, apoptosis was increased in MORC2 p.Thr424Arg (199%), p.Gly444Arg (239%), p.His446Gln (235%) compared to WT MORC2 (100%) electroporated neurons.

### 3.6 MORC2 affect cortical neuron neurite outgrowth

Axonal forms of CMT and SMA affect axonal growth or maintenance in patients. To investigate the impact of MORC2 mutations on the axonal compartment, we evaluated neurite outgrowth in neurons electroporated with the eGFP reporter gene (Figure 4c). Box plot of the results showed a significant reduction of neurite outgrowth in neurons expressing p.Thr424Arg (mean: 87.96), p.Gly444Arg (53.37) and p.His446Gln (66.43) compared to neurons expressing WT MORC2 (107.85). Altogether, these results show that MORC2 mutants interfere with neurite outgrowth, suggesting that they cause axonal stress. This supports the conclusion drawn from survival and apoptosis experiments performed in SH‐EP and motor neurons that indicated that the p.Gly444Arg and p.His446Gln variants of unknown significance are probably pathogenic.

## 4 DISCUSSION

Here we report the use of in vitro models to assess the pathogenicity of variants of unknown significance in the *MORC2* gene. To this end, we have used the human neuroblastoma cell line SH‐EP which is a proliferative and easy to transfect cell line, and the primary cortical neurons which are abundant and easy to electroporate. We found that in SH‐EP line the overexpression of a pathogenic MORC2 mutant significantly decreased survival and increased apoptosis. The same effect was observed in primary cortical neurons in which we found that neurite outgrowth was also significantly reduced.

A previous study performed in rat sensory neurons showed that the pSer87Leu mutation in *MORC2* which is associated with a severe SMA like phenotype caused axonal swelling (Sancho et al., 2019). To date, only one in vitro cell model has been developed to establish the pathogenicity of de novo variants of the *MORC2* gene (Guillen Sacoto et al., 2020). This model is based on the ability of MORC2 to regulate the activity of the HUSH complex involved in epigenetic silencing (Liu et al., 2017; Tchasovnikarova et al., 2017). This approach was performed on a population of FACS‐isolated HeLa cells that displayed epigenetic repression of an integrated GFP reporter by the HUSH complex. The knockout of the *MORC2* gene de‐repressed the GFP reporter gene and the ability of transfected MORC2 constructs to restore repression GFP repression by the HUSH complex was evaluated by FACS (Douse et al., 2018; Guillen Sacoto et al., 2020; Tchasovnikarova et al., 2017). Altogether, these studies provided a precise evaluation of the regulatory function of MORC2 mutants in the HUSH complex but did not consider possible other biological functions and revealed the necessity to develop simple in vitro models applicable to the analysis of many variants in the context of hospital facilities to help clinicians to quickly investigate new variants and establish molecular diagnosis.

We have identified two different mutations in the *MORC2* gene in patients from two unrelated families. Patients from the first family presented a form of young adult‐onset, autosomal dominant sensorimotor axonal neuropathy with progressive weakness and mild sensory impairment. The overall clinical manifestations of the present patients resemble those in the original paper by Sevilla et al. describing the p.Arg252Trp and p.Ser87Leu mutations, but with a less prominent sensory loss at neurological examination and a slightly later age of onset. Some patients have been identified with early, SMA‐like disease onset and primary involvement of the proximal muscles (Albulym et al., 2016; Laššuthová et al., 2016; Schottmann et al., 2016; Sevilla et al., 2016). Patient identified in the second family present with late proximal motor neuropathy without any sign of sensory nerve involvement either from clinical or electrophysiological point of view. Although the involvement of the sensory nerves was an initial and prominent feature, especially in patients with SMA‐like phenotypes (Sevilla et al., 2016), some studies reported minor or even none sensory complaints (Albulym et al., 2016; Laššuthová et al., 2016; Schottmann et al., 2016). The main clinical characteristic of *MORC2* mutated patients is the spreading of muscle weakness to proximal muscles. It is observed in the majority of patients and leads to severe weakness and walking difficulties (Semplicini et al., 2017; Sevilla et al., 2016). In some patients, the clinical phenotype tends to be more complex since it may include symptoms of the central nervous system, which comprise pyramidal signs, neurodevelopmental disorders with growth retardation and cerebellar atrophy (Albulym et al., 2016; Guillen Sacoto et al., 2020; Schottmann et al., 2016). Altogether, *MORC2* is associated with a broad genetic and phenotypic variability that is challenging for neurologists (Jacquier et al., 2022; Sivera et al., 2021). Interestingly, apart from *SMN* only a few genes can cause SMA‐like phenotypes in adults: *TRPV4, CHCHD10; BICD2, DYNHC1H1, SETX, VRK1, VAPB, CAPN1, and ASAH1* genes have been reported (Peeters et al., 2014; Perez‐Siles et al., 2021). Our study identifies a new variant of *MORC2* gene associated with a late‐onset proximal motor neuropathy, resembling SMA phenotype, thus increasing the number of patients with such phenotype. This suggests that *MORC2* should be included not only in the panel of genes involved in CMT but also investigated in general in patients proximal motor neuropathy. To some extent, considering the broad range of phenotypes associated with *MORC2* mutations, this gene should also be considered for other panels such as spinocerebellar ataxia, or neurodevelopmental disorders.

## CONFLICT OF INTEREST

The authors declare no conflict of interest.

## Supporting information

Supporting information.
