# Supplementary material for: Expanding the phenotypic variability of MORC2 gene mutations: From Charcot‐Marie‐Tooth disease to late‐onset pure motor neuropathy
Source: Hum Mutat. 2022 Aug 18;43(12):1898–908. doi: 10.1002/humu.24445 (PMC10087860; doi:10.1002/humu.24445)
Supplement: Supplementary file 1 — Supporting information. [file HUMU-43-1898-s001.docx]

**Supp. Figure S1**


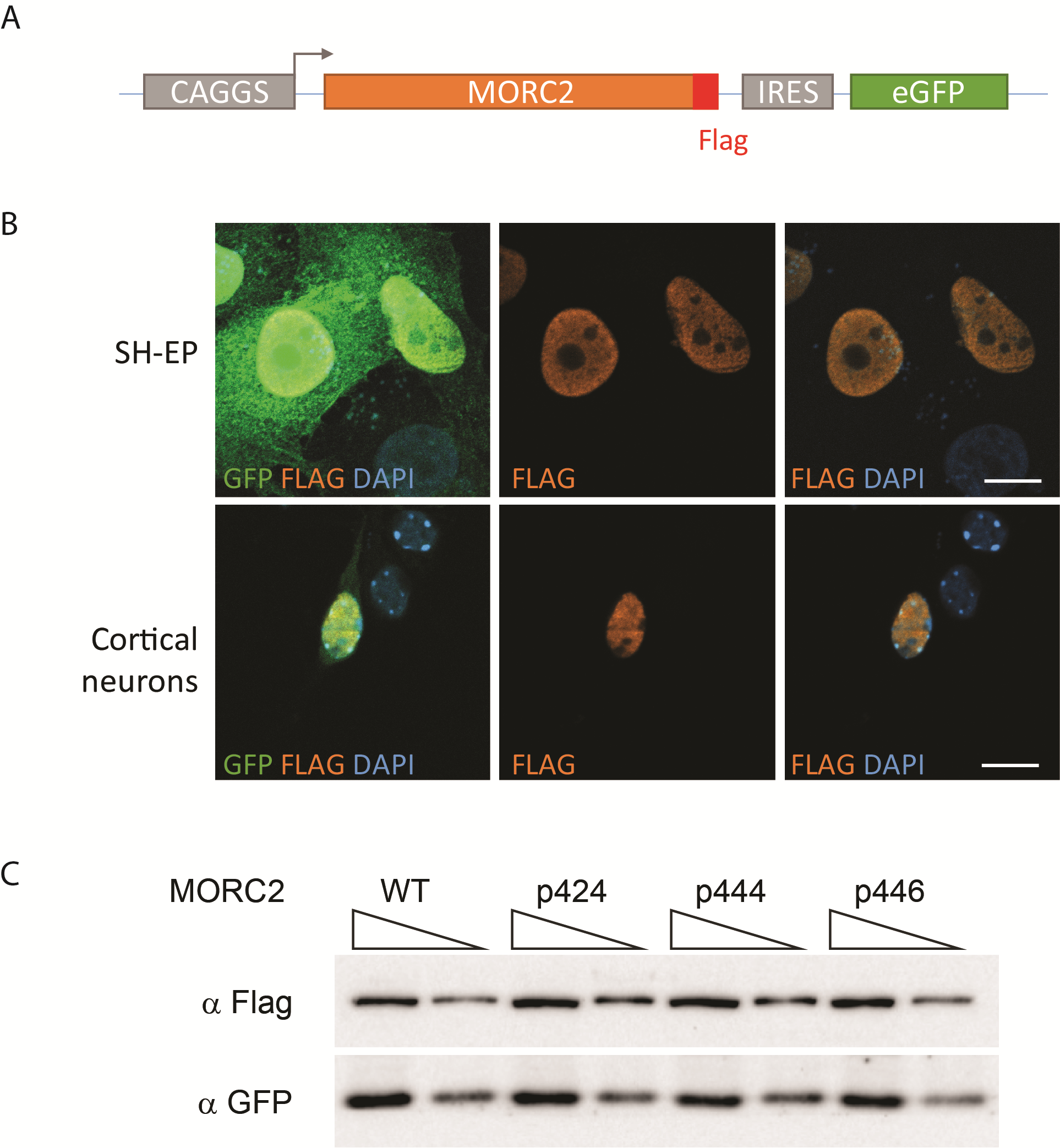


A. Representation of the plasmid construct based on pCAGIG vector with a CAGG promotor and an IRES allowing the transduction of eGFP and MORC2. MORC2 is tagged by Flag epitope in C-terminal. B. Confocal images of SH-EP or cortical neuron transfected with WT MORC2 construct. Cells were counter-stain with DAPI and anti-Flag to show nuclei and MORC2 subcellular localization, respectively. C. MORC2 quantification by Western Blot of SH-EP transfected by the plasmid and lysed at 1 day after transfection. Volume 1x and 2x were loaded for each condition.
